# Physical education class participation is associated with physical activity among adolescents in 65 countries

**DOI:** 10.1038/s41598-020-79100-9

**Published:** 2020-12-17

**Authors:** Riaz Uddin, Jo Salmon, Sheikh Mohammed Shariful Islam, Asaduzzaman Khan

**Affiliations:** 1 Institute for Physical Activity and Nutrition (IPAN), School of Exercise and Nutrition Science, Deakin University, Geelong, VIC 3220 Australia; 2 School of Health and Rehabilitation Sciences, The University of Queensland, Therapies Annex, St Lucia, Brisbane, QLD 4072 Australia; 3 Active Healthy Kids Bangladesh (AHKBD), Dhaka, Bangladesh

**Keywords:** Health services, Public health, Paediatric research

## Abstract

In this study we examined the associations of physical education class participation with physical activity among adolescents. We analysed the Global School-based Student Health Survey data from 65 countries (N = 206,417; 11–17 years; 49% girls) collected between 2007 and 2016. We defined sufficient physical activity as achieving physical activities ≥ 60 min/day, and grouped physical education classes as ‘0 day/week’, ‘1–2 days/week’, and ‘ ≥ 3 days/week’ participation. We used multivariable logistic regression to obtain country-level estimates, and meta-analysis to obtain pooled estimates. Compared to those who did not take any physical education classes, those who took classes ≥ 3 days/week had double the odds of being sufficiently active (OR 2.05, 95% CI 1.84–2.28) with no apparent gender/age group differences. The association estimates decreased with higher levels of country’s income with OR 2.37 (1.51–3.73) for low-income and OR 1.85 (1.52–2.37) for high-income countries. Adolescents who participated in physical education classes 1–2 days/week had 26% higher odds of being sufficiently active with relatively higher odds for boys (30%) than girls (15%). Attending physical education classes was positively associated with physical activity among adolescents regardless of sex or age group. Quality physical education should be encouraged to promote physical activity of children and adolescents.

## Introduction

Physical activity is essential for health and wellbeing of children and adolescents^1^. Physical activity improves musculoskeletal, cardiac, metabolic, psychosocial, and cognitive health, and enhances cardiorespiratory and muscular fitness of children and adolescents^1–4^. Regular participation also decreases adiposity in those who are overweight^3^. For optimal health benefits, the current international guidelines (i.e., the World Health Organization [WHO]) recommends that those aged 5–17-years accumulate at least 60 min of moderate-to-vigorous physical activity daily^5^. Globally, four out of five (81%) adolescents aged 11–17 years do not meet this recommendation and are insufficiently active^6^. Such inactive behaviours during adolescence have both current and future ramifications on health and wellbeing as behaviours such as physical activity established during adolescence can carry over to adulthood^7,8^. Therefore, pragmatic strategies to promote physical activity during adolescence around the globe are of critical importance^9^.

Adolescent physical activity occurs in different settings and domains including at home, in the community, for transportation, and at school. Opportunities for physical activity at school include during recess and lunch breaks, school sport and physical education lessons. Physical education classes may provide resources and opportunities for students to accumulate the daily physical activity level and can contribute to daily energy expenditure^10,11^. Recent meta-analyses found that 41% of secondary school^12^ and 45% of elementary school^13^ physical education lessons comprised moderate-to-vigorous physical activity. In many countries, physical education provides children and adolescents the understanding and motivation for an active lifestyle and also creates an environment to acquire knowledge and skills for physical activity throughout life^14,15^. In addition, adolescents who may have limited access to space and equipment outside of school can benefit from attending physical education classes at school^10,11^. School-based physical education, therefore, can be an accessible source of physical activity for many adolescents and can help develop an active healthy lifestyle^16^. In addition to the number of physical education classes, access to high-quality physical education experience (e.g., teacher behaviours, learning outcomes), which forms the foundation for lifelong engagement in physical activity, is also important for children and adolescents^17–19^.

Available evidence suggest that participation in physical education classes are positively associated with higher levels of physical activity^20–24^. However, the evidence is mostly based on single-country studies from high-income countries with limited multi-country study and lack of representation of low- and lower-middle-income countries^25^. A recent multi-country study reported country- and regional-level differences in physical education class participation, which was also differed by sex, age, and country-income classification^26^. In addition, delivery, content and quality of physical education also vary within and between countries^27,28^. It is often provided infrequently in schools across countries, and therefore the potential impact on total moderate-to-vigorous physical activity among boys and girls may be limited^29^. In order to obtain a comprehensive global perspective on the relationship between physical education and physical activity, large multi-country studies with representative samples are essential. Given the context and the opportunities that exist in schools for physical activity promotion, in this study, we aimed to examine whether participation in physical education classes (i.e., number of physical education class attendance) is associated with sufficient level of physical activity among adolescents (overall, and by sex and age-group) from 65 countries around the globe. We hypothesised that higher number of physical education class participation would be positively associated with sufficient level of physical activity among adolescents.

## Methods

### Data source

Data for this study were from the Global School-based Student Health Survey (GSHS), a population-based survey of school-going children and adolescents around the world^30^. In all participating countries, the GSHS uses the same standardised sampling technique and study methodology. All participants completed a standardised self-administered anonymous questionnaire, which included, but was not limited to, questions on demographics (e.g., age, sex), participation in physical education classes and physical activity. GSHS adopted questionnaire items, including items to measure physical activity and physical education from the Youth Risk Behavior Survey of American Adolescents. Countries, where GSHS were implemented, were encouraged to use culturally appropriate examples, words, and phrases to ensure sociocultural adaptability of the items. Furthermore, using a rigorous translation and back-translation process with the assistance of WHO and US CDC, countries were allowed to translate the questionnaire into their local language^31^.

As of 8 December 2019, 98 countries/territories around the globe had at least one GSHS dataset publicly available with the surveys being conducted between 2007 and 2016. For countries with more than one GSHS dataset, we used the most recent one available. Of the 98 countries, 84 countries had data on PA, while 67 countries had data on physical education. Two countries (Niue and Tokelau) were excluded from the analyses due to their small sample size (n < 140). The analytical sample consists of 206,417 adolescents aged 11 or younger to 17 years from 65 countries. Only a small proportion of students (1.05%) were in the age group “11 years old or younger”, and for modelling purposes, they were considered as 11 years old for this analysis, as it was not possible to determine what proportion of 1.05% students were younger than 11 years old. All countries provided nationally representative samples.

The GSHS received ethics approval from the Ministry of Education or a relevant Institutional Ethics Review Committee, or both in each of the participating countries. Only those adolescents and their parents who provided written or verbal consent participated. As the current study used retrospective, de-identified, publicly available data, ethics approval was not required for this secondary analysis. Detailed methods of the GSHS have been described on both the US CDC and the WHO websites^30,32^.

### Outcome measure—physical activity participation

Physical activity was assessed with one item: ‘During the past 7 days, on how many days were you physically active for a total of at least 60 min per day?’ The response options were 0–7 days. Consistent with the WHO recommendations^5^, we defined participants as ‘sufficiently active’ who did ≥ 60 min/day of physical activity on seven days of the week.

### Study factor—physical education participation

Physical education class attendance was assessed with one item: ‘During this school year, on how many days did you go to physical education (PE) class each week?’ The responses were classified into three groups: ‘0 day/week’, ‘1–2 days/week’, and ‘≥ 3 days/week’ as used elsewhere^25,33^.

### Covariates

Adolescents self-reported age, sex, and daily hours of sitting (when not in school or doing homework) in the survey. Food insecurity was assessed by asking: ‘During the past 30 days, how often did you go hungry because there was not enough food in your home?’ with response options being never, rarely, sometimes, most of the time, and always. As the GSHS did not include any direct measure of socioeconomic status, this variable was used as a proxy measure of socioeconomic status^34,35^. Self-reported height and weight were used to compute body mass index (BMI), which was categorised as underweight (BMI < −2SD), overweight (BMI >  + 1SD), and obese (BMI >  + 2SD), relative to median BMI, by age and sex based on the WHO Child Growth Standards^36^.

### Statistical analyses

Of the 65 countries with data on physical activity and physical education, nine countries were from Africa, 20 from the Americas, 15 from Eastern Mediterranean, five from South East Asia, and 16 from the Western Pacific region. Using the World Bank country classification, collected at the time of the survey for the respective countries, seven countries were classified as low‐income, 21 lower‐middle‐income, 18 upper‐middle‐income, and 18 high-income. Income classification information was not available for Cook Island. The prevalence estimates of physical activity and physical education were obtained by using a Stata command ‘svyset’ to take into account sampling weights and the clustered sampling design of the surveys.

In examining the country-level association of physical education with physical activity, a set of covariates was considered including age, sex, weight status (i.e., BMI), food insecurity, and sitting time. Sitting time was considered as an adjusting factor given its demonstrated association with physical activity in adolescents^37^. Given the binary nature of physical activity outcome, logistic regression analysis with robust standard errors was used to examine the association at the country level, by taking into account the sampling weight that was applied to each participant record to adjust for non-response and the varying probability of selection. This GSHS weighting factor was applied in an identical way to estimate the association in each participating country. Within the GSHS protocol, weighting accounted for the probability of selection of schools and classrooms, non-responding schools and students, and distribution of the population by sex and grade.

Random effects meta-analysis was used to generate pooled estimates of the association between physical education and physical activity for the overall sample, by country income category (e.g., low-income, lower-middle income, upper-middle income, and high-income), and by WHO region, stratified by sex and age groups (11–14 years vs 15–17 years). Two age groups (11–14 years [early adolescence] and 15–17 years [middle adolescence])^38^ were considered to stratify the analysis in order to examine whether the association estimates vary across phases of adolescence. This analysis used DerSimonian and Laird method^39^ with the estimate of heterogeneity being taken from the Mantel–Haenszel model. As the GSHS were conducted across different cultural settings in 65 countries around the world over a long period of time (2007–2016), it was reasonable to assume that the association estimates across countries were likely to vary from survey to survey, which supports the use of random effects meta-analysis that can adjust heterogeneity among studies^40^. The percentage of variability in estimates across studies that is attributable to between study heterogeneity (I^2^) in our analysis ranges from 54.3 to 80.2%, which suggests a strong presence of heterogeneity in the association estimates, and further supports the use of random effects meta-analysis. All adjusted estimates of the association parameters are presented in the form of odds ratio (OR) and 95% confidence interval (CI). All analyses were conducted by StataSE V14.0.

### Ethics approval and consent to participate

The GSHS received ethics approval from both a national government administration and an institutional review board or ethics committee. Only adolescents and their parents who provided written/verbal consent participated. As the current study used retrospective publicly available data, we did not require ethics approval from any Institutional Ethics Review Committee for this secondary analysis.

## Results

**Figure 1:**
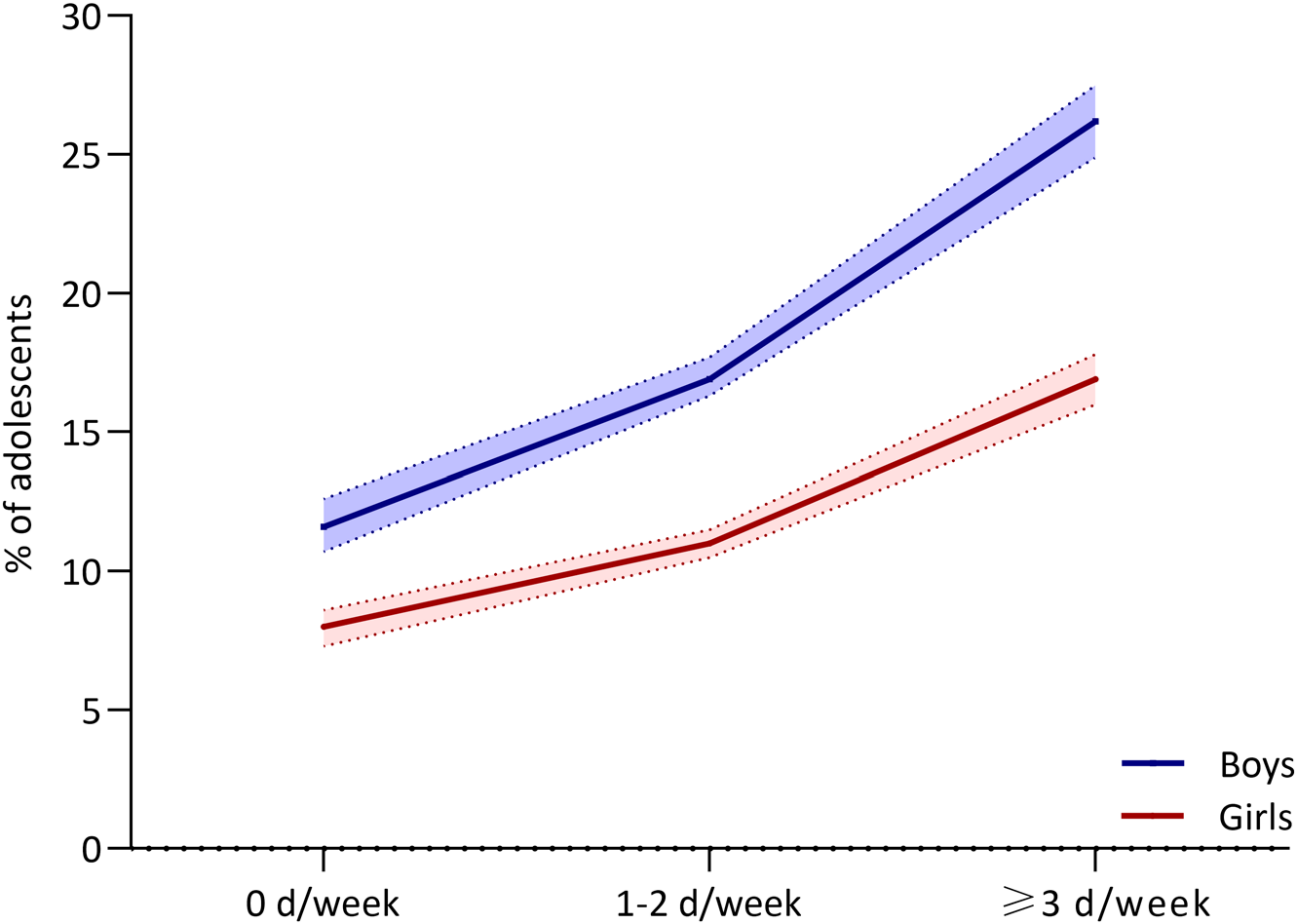
Proportion of adolescents sufficiently physically active by participation in physical education classes, Global School-based Student Health Survey, 2007–2016.

The mean age of the participating adolescents (n = 206,417) was 14.35 (SD = 1.45) years, 54.4% aged 11–14 years, and 49.2% were girls. The prevalence of sufficient physical activity was 15.0%, with boys having higher prevalence (18.3%) than girls (11.5%). Over half (56.5%) of adolescents participated in physical education classes 1–2 days/week (boys 54.7%; girls 58.3%) and about a quarter (24.2%) participated in physical education classes ≥ 3 days/week (boys 26.8%; girls 21.6%). As shown in Fig. 1, the overall percentage of adolescents being sufficiently active was greater for those who attended more physical education classes in both sexes.

Estimates of associations of physical education class participation with sufficient physical activity by country are shown in Table 1. The country-level analysis shows that 50 out of 65 participating countries (77%) demonstrated significant and positive associations between attending physical education classes ≥ 3 days/week and being sufficiently active with 33 countries (51%) revealing at least double the odds (OR ≥ 2.0) of meeting physical activity guidelines. For example, Bolivian adolescents who attended physical education classes ≥ 3 days/week had threefold odds of reporting sufficient physical activity compared with their counterparts who attended no physical education class (OR 3.00, 95% CI 1.93–4.67). In examining the association between attending physical education classes 1–2 days/week and being sufficiently active, 20 countries (31%) demonstrated significant positive associations. For example, Thai adolescents who attended physical education classes 1–2 days/week had double the odds to reporting sufficient physical activity compared with their counterparts who attended no physical education class (OR 2.11, 95% CI 1.39–3.19). As shown in Table 1, attending physical education classes ≥ 3 days/week was positively and strongly associated with physical activity in all WHO regions with South East Asia region showing the strongest association (OR 2.89, 2.11–3.97), followed by Africa (OR 2.45, 1.72–3.48) and Western Pacific region (OR 2.40, 1.92–3.00). The analysis also showed evidence of positive and moderate association between attending physical education classes 1–2 days/week and being sufficiently active in all WHO regions with the pooled association estimates ranging from OR 1.19 (1.01–1.41) in the Americas region to OR 1.86 (1.03–3.36) in South East Asia.

**Table 1:** Country-level adjusted^a^ and region-level pooled estimates of association between frequency of physical education classes and sufficient physical activity among adolescents aged 11–17 years, Global School-based Student Health Surveys, 2007–2016.

Region/country (n)	Physical education classes ≥ 3 days/week and physical activity	Physical education classes 1–2 days/week and physical activity
	OR (95% CI)	OR (95% CI)
Africa (n = 21,797)	2.45 (1.72–3.48)	1.28 (1.11–1.48)
Algeria (4228)	2.36 (1.70–3.28)	1.00 (0.73–1.36)
Benin (1503)	2.07 (1.24–3.46)	1.14 (0.76–1.72)
Ghana (2264)	1.72 (1.21–2.45)	1.16 (0.82–1.63)
Mauritania (1249)	2.61 (1.69–4.04)	1.09 (0.65–1.82)
Mauritius (3002)	1.99 (1.26–3.16)	1.21 (0.78–1.88)
Mozambique (1245)	2.05 (0.84–5.01)	1.81 (0.81–4.04)
Namibia (3013)	1.66 (1.22–2.27)	1.66 (1.24–2.23)
Seychelles (1965)	3.14 (2.07–4.76)	1.79 (1.18–2.71)
Tanzania (3328)	5.92 (4.62–7.59)	1.23 (0.90–1.67)
The Americas (n = 53,477)	1.50 (1.27–1.77)	1.19 (1.01–1.41)
Antigua & Barbuda (1103)	0.98 (0.65–1.50)	0.76 (0.52–1.11)
Argentina (16,390)	1.55 (1.10–2.19)	1.02 (0.73–1.41)
Bahamas (1119)	5.46 (1.76–16.91)	5.22 (1.73–15.77)
Barbados (1253)	1.31 (0.81–2.09)	0.94 (0.59–1.49)
Belize (1826)	1.34 (0.98–1.85)	0.93 (0.71–1.22)
Bolivia (3209)	3.00 (1.93–4.67)	2.12 (1.37–3.26)
British Virgin Island (1433)	1.36 (0.91–2.03)	1.36 (0.95–1.95)
Chile (1681)	1.79 (0.94–3.38)	1.15 (0.61–2.17)
Costa Rica (2521)	1.71 (1.23–2.36)	1.20 (0.87–1.65)
Curaçao (2013)	2.17 (1.31–3.59)	1.50 (0.91–2.48)
Dominica (1181)	2.32 (1.41–3.82)	1.43 (0.90–2.27)
El Salvador (1665)	1.02 (0.63–1.65)	1.06 (0.66–1.68)
Guatemala (3491)	4.64 (1.80–11.96)	4.11 (1.64–10.28)
Guyana (2160)	0.90 (0.64–1.27)	0.72 (0.54–0.97)
Honduras (1583)	0.87 (0.50–1.51)	0.96 (0.57–1.62)
Peru (2603)	2.47 (0.90–6.77)	2.50 (1.46–4.29)
Saint Kitts and Nevis (1401)	0.92 (0.61–1.39)	1.13 (0.81–1.56)
Suriname (1483)	1.61 (1.11–2.32)	1.32 (0.96–1.81)
Trinidad and Tobago (2359)	1.56 (1.10–2.22)	1.21 (0.86–1.69)
Uruguay (3003)	1.23 (0.88–1.72)	0.64 (0.46–0.89)
Eastern Mediterranean (n = 46,944)	2.01 (1.72–2.35)	1.21 (1.04–1.40)
Afghanistan (1599)	1.11 (0.73–1.68)	0.62 (0.38–1.01)
Bahrain (6724)	2.25 (1.81–2.81)	1.36 (1.10–1.69)
Egypt (2028)	1.21 (0.81–1.82)	0.76 (0.56–1.04)
Iraq (1756)	2.44 (1.72–3.48)	1.12 (0.76–1.64)
Kuwait (2603)	3.55 (2.30–5.49)	2.37 (1.62–3.45)
Lebanon (1444)	1.90 (1.34–2.68)	1.09 (0.74–1.59)
Morocco (2525)	2.33 (1.67–3.25)	0.88 (0.62–1.25)
Palestine (10,350)	2.17 (1.80–2.63)	1.41 (1.17–1.71)
Oman (2807)	1.63 (1.16–2.27)	1.03 (0.70–1.52)
Pakistan (4793)	1.33 (1.02–1.72)	1.29 (1.05–1.59)
Qatar (1609)	3.62 (2.15–6.07)	2.29 (1.37–3.82)
Sudan (1806)	1.74 (1.06–2.87)	1.10 (0.72–1.69)
Syria (2847)	2.88 (1.91–4.33)	1.37 (0.92–2.04)
UAE, The (2185)	1.70 (1.25–2.32)	1.17 (0.87–1.56)
Yemen (1868)	2.43 (1.71–3.46)	1.28 (0.84–1.94)
South East Asia (n = 25,401)	2.89 (2.11–3.97)	1.86 (1.03–3.36)
Bangladesh (2446)	2.86 (1.79–4.59)	2.93 (1.83–4.70)
Indonesia (9992)	3.33 (2.42–4.57)	2.37 (1.78–3.14)
Nepal (5350)	1.94 (1.58–2.38)	0.71 (0.54–0.94)
Thailand (5193)	3.26 (1.99–5.33)	2.11 (1.40–3.19)
Timor Leste (2420)	4.30 (2.34–7.90)	2.32 (1.28–4.20)
Western Pacific (n = 58,798)	2.40 (1.92–3.00)	1.24 (1.05–1.46)
Brunei (2378)	1.67 (1.14–2.43)	0.78 (0.55–1.12)
Cambodia (2715)	6.52 (3.68–11.57)	1.77 (1.09–2.89)
Cook Island (602)	2.30 (1.26–4.21)	0.83 (0.42–1.67)
Fiji (2674)	1.43 (1.04–1.96)	1.37 (1.04–1.79)
Kiribati (1389)	3.88 (2.46–6.12)	1.13 (0.70–1.83)
Laos (3449)	1.74 (1.22–2.48)	1.22 (0.95–1.57)
Malaysia (24,215)	2.12 (1.79–2.51)	1.37 (1.17–1.61)
Mongolia (4950)	4.13 (2.48–6.86)	2.72 (1.72–4.29)
Philippines, The (6918)	1.60 (1.12–2.28)	1.29 (0.89–1.87)
Samoa (1385)	2.94 (1.94–4.47)	1.08 (0.68–1.70)
Solomon Islands (1061)	1.96 (1.21–3.19)	1.67 (0.98–2.82)
Tonga (1988)	2.68 (1.98–3.64)	0.74 (0.51–1.07)
Tuvalu (542)	4.22 (2.33–7.66)	0.53 (0.19–1.48)
Vanuatu (763)	0.50 (0.20–1.26)	1.27 (0.71–2.28)
Vietnam (2878)	1.76 (0.69–4.47)	1.10 (0.50–2.42)
Wallis & Futuna (891)	8.14 (2.96–22.43)	2.69 (1.03–7.01)

*OR* odds ratio, *CI* confidence intervals, *UAE* United Arab Emirates.

^a^Reference being attending no physical education class. Adjusted for age, sex, weight status (i.e., BMI), food insecurity, and sitting time.

Overall, adolescents who took physical education classes ≥ 3 days/week, compared to those who did not take any physical education classes, had double the odds of being sufficiently active (OR 2.05, 95% CI 1.84–2.28) with no apparent gender (OR 2.09, 1.88–2.33 for boys; and OR 1.95, 1.69–2.25 for girls) or age (OR 2.19, 1.93–2.48 for 11–14-year-old; and OR 2.03, 1.80–2.28 for 15–17-year-old adolescents) differences (Table 2). Adolescents who participated in physical education classes 1–2 days/week had 26% higher odds of being sufficiently active (OR 1.26, 1.15–1.37) with relatively higher odds for boys (OR 1.30, 1.17–1.46) than girls (OR 1.15, 1.03–1.29) and younger adolescents aged 11–14 years (OR 1.28, 1.16–1.42) that older adolescents aged 15–17 years (OR 1.19, 1.08–1.32).

**Table 2:** Pooled estimates of association between frequency of physical education classes and sufficient physical activity among adolescents aged 11–17 years, by World Bank *country income classification*, Global School-based Student Health Surveys, 2007–2016.

Category	Physical education classes ≥ 3 days/week and physical activity	Physical education classes 1–2 days/week and physical activity
	OR^a^ (95% CI)	OR^a^ (95% CI)
**Total**		
Low-income (n = 17,740)	2.37 (1.51–3.73)	1.06 (0.82–1.37)
Lower-middle income (n = 70,453)	2.09 (1.71–2.56)	1.39 (1.19–1.62)
Upper-middle income (n = 64,705)	2.02 (1.71–2.39)	1.23 (1.05–1.43)
High-income (n = 52,917)	1.85 (1.52–2.25)	1.25 (1.04–1.49)
Overall (n = 206,417)^b^	2.05 (1.84–2.28)	1.26 (1.15–1.37)
**Boys**		
Low-income (n = 9757)	2.51 (1.70–3.70)	1.03 (0.71–1.49)
Lower-middle income (n = 37,009)	2.15 (1.77–2.60)	1.46 (1.21–1.76)
Upper-middle income (n = 33,903)	2.09 (1.78–2.46)	1.33 (1.10–1.62)
High-income (n = 27,907)	1.89 (1.50–2.37)	1.27 (1.03–1.55)
Overall (boys) (n = 108,879)^b^	2.09 (1.88–2.33)	1.30 (1.17–1.46)
**Girls**		
Low-income (n = 7983)	2.36 (1.31–4.26)	1.12 (0.75–1.67)
Lower-middle income (n = 33,444)	2.07 (1.58–2.72)	1.30 (1.03–1.65)
Upper-middle income (n = 30,802)	1.83 (1.45–2.32)	1.06 (0.93–1.20)
High-income (n = 25,010)	1.69 (1.36–2.10)	1.09 (0.87–1.35)
Overall (girls) (n = 97,538)^b^	1.95 (1.69–2.25)	1.15 (1.03–1.29)
**11–14 years**		
Low-income (n = 8851)	2.94 (1.92–4.51)	1.21 (0.91–1.61)
Lower-middle income (n = 37,637)	2.23 (1.77–2.82)	1.36 (1.09–1.68)
Upper-middle income (n = 31,631)	2.21 (1.83–2.68)	1.26 (1.07–1.49)
High-income (n = 28,555)	1.83 (1.47–2.28)	1.22 (1.03–1.46)
Overall (11–14 years) (106,868)^b^	2.19 (1.93–2.48)	1.28 (1.16–1.42)
**15–17 years**		
Low-income (n = 8889)	2.32 (1.36–3.96)	0.99 (0.74–1.33)
Lower-middle income (n = 32,816)	2.17 (1.76–2.68)	1.33 (1.16–1.51)
Upper-middle income (n = 33,074)	1.98 (1.61–2.44)	1.18 (0.98–1.43)
High-income (n = 24,362)	1.80 (1.48–2.19)	1.14 (0.91–1.44)
Overall (15–17 years) (n = 99,549)^b^	2.03 (1.80–2.28)	1.19 (1.08–1.32)

^a^Reference being attending no physical education class.

^b^Cook Island (n = 602), which was not a part of income classification, was included in calculating the overall pooled estimate. *OR* odds ratio, *CI* confidence intervals.

The odds of attending physical education classes ≥ 3 days/week and being sufficiently active were lower in country with higher income (Table 2). In low-income countries, adolescents who participated in physical education classes ≥ 3 days/week had 137% higher odds of being sufficiently active (OR 2.37, 1.51–3.73) with comparable odds for boys (OR 2.51, 1.70–3.70) and girls (OR 2.36, 1.31–4.26) and slightly higher odds for younger (OR 2.94, 1.92–4.51) than older adolescents (OR 2.32, 1.36–3.96). In high-income countries, the odds of being sufficiently active was 85% higher for adolescents who attended physical education classes ≥ 3 days/week (OR 1.85; 1.52–2.25) with no apparent gender (boys OR 1.89, 1.50–2.37; girls OR 1.69, 1.36–2.10) or age (younger OR 1.83, 1.47–2.28; older OR 1.80 (1.48–2.19) differences. In lower-middle income countries, adolescents who attended physical education classes 1–2 days/week had 39% higher odds of being sufficiently active (OR 1.39, 1.19–1.62) compared to their counterparts who did not take any physical education classes, with relatively higher odds for boys (OR 1.46, 1.21–1.76) than girls (OR 1.30, 1.03–1.65), and similar odds for younger (OR 1.36, 1.09–1.68) and older adolescents (OR 1.33, 1.16–1.51).

Boys of South East Asian region who participated in physical education classes ≥ 3 days/week had the highest odds of being sufficiently active (OR 3.29, 1.97–5.47), followed by the boys of Africa region (OR 2.41, 1.74–3.33) (Supplementary Table S1). Girls of Western Pacific and Africa region who participated in physical education classes ≥ 3 days/week had the highest odds of being sufficiently active (OR 2.68, 1.89–3.77, and OR 2.63, 1.63–4.26, respectively). Even by attending physical education classes 1–2 days/week, boys of the Americas region and girls of Africa region can increase their odds, though not considerably, of being sufficiently active (OR 1.29, 1.06–1.58, and OR 1.41, 1.15–1.73, respectively).

Both younger and older adolescents in all WHO regions demonstrated positive association between ≥ 3 days/week physical education class attendance and meeting the physical activity recommendations (Supplementary Table S1). Younger adolescents in South East Asia (OR 3.03, 2.42–3.79) and Africa (OR 2.95, 2.07–4.20), and older adolescents in South East Asia (OR 3.24, 1.57–6.67) who participated in physical education classes ≥ 3 days/week had over three times higher odds of being sufficiently active. There were moderate positive associations between physical education class attendance for 1–2 days/week and meeting the physical activity recommendations for younger adolescents in Africa (OR 1.38, 1.03–1.84), the Americas (OR 1.29, 1.07–1.56), and Eastern Mediterranean regions (OR 1.24, 1.06–1.44), and for older adolescents in Africa (OR 1.24, 1.03–1.48), Eastern Mediterranean (OR 1.26, 1.07–1.49), and Western Pacific region (OR 1.19, 1.01–1.41).

## Discussion

To our knowledge, this is the most extensive global study to assess the association of physical education class attendance with physical activity of adolescents, based on nationally representative samples from 65 countries around the globe. The key finding of our study is that adolescents, irrespective of sex or age, who had a higher frequency (≥ 3 days/week) of physical education class attendance had significantly higher odds of meeting the WHO’s physical activity recommendations. The estimates of association between the frequency of attending physical education and meeting physical activity recommendations were lower among countries with higher income. We observed some regional differences with South East Asia having the highest associations and the Americas having the lowest. Our findings suggest that adolescents, especially girls and those aged 15–17 years, are mostly benefited from a higher frequency (i.e., ≥ 3 days/week) of physical education participation. Our study also found some benefits of less frequent participation in physical education classes (1–2 days/week) in meeting the physical activity guidelines, which is encouraging. About one-third of the countries demonstrated positive association between less frequent participation in physical education classes and meeting the physical activity recommendations, and such association was prominent in boys and younger adolescents in all but low-income countries. Our study thus argues that even less frequent participation in physical education classes can bring some benefits for some adolescents.

Our finding that a higher frequency of physical education class attendance was positively associated with meeting the physical activity recommendations is consistent with other studies in children and adolescents^20,21,24,25^. It has been argued that participation in physical education classes acts as a positive reinforcement to “keep young people going” by being more physically active with less time in sedentary behaviour throughout the day^25^. Physical education classes provide children with an opportunity to familiarise themselves with different types of physical activity, motivates them to be active within the school environment, and potentially also encourages more out-of-school physical activity^41^. Physical activity during physical education classes may reduce fatigue and improve mood by changing neurophysiological stimulation and the brain’s information processing function (i.e., cerebral cortex), which may improve children’s preparedness to move more throughout the day^25^. While the frequency of physical education class is important, it is also critical that children have access to quality physical education^18,19^. Previously, researchers have suggested that in spite of the traditional class-based and sports-centred physical education curriculum, physical education ought to be a health-centred dynamic learning experience for children^19,42^. Quality physical education is important for age-appropriate cognitive learning and to acquire fitness, develop motor skills and psychosocial and emotional skills, which can help children to lead an active lifestyle, inside and outside of the school environment, throughout their life course^18,19,42^. Given the role of physical education for active and healthy lifestyle, different stakeholders, including United Nations agencies (i.e., UNESCO)^19^, European Commission^17^, have recommended to ensure quality physical education for children and adolescents, and called for political commitments and actions from Governments and supports from the international communities.

In our study, adolescents boys and girls in low-income countries with ≥ 3 days/week physical education class attendance had the highest odds of meeting the physical activity recommendations, and the associations became smaller (yet significant) with a higher country income classification for both sexes. A previous 12-country study^25^ reported similar findings for boys, but not for girls. Unlike our study that is based on self-reported data, the earlier study used a device-based physical activity measure and included Australia and other high-income countries of Europe and North America. In addition to high-income countries, our study included adolescents from low- and lower-middle-income countries. It is possible that for many children, regardless of sex or country income, schools provide the most pragmatic and readily accessible opportunities for various physical activity, while out-of-school physical activity options, logistics, and environments might be variable^10,11^. The environments, in general, may be more supportive of out of school physical activity for children in high-income countries than their counterparts in low-income countries; however, high-income countries may have other challenges including gender and socioeconomic disparities in physical activity. For example, children from high-poverty neighbourhood may have fewer opportunities for out of school physical activity in many high-income countries^43,44^. Appreciating the heterogeneity in resources for physical education within- and across countries, all governments should consider schools as the primary focus to promote an active and healthy lifestyle among children and adolescents, which is likely to be a cost-effective and opportunistic initiative to get them moving. Our findings also show that physical education is potentially more important in South East Asia than the Americas in promoting physical activity. In addition to environmental support, such variations could be a sign of the quality of the respective physical education programs, including time allocated for physical education across the countries. There is a large heterogeneity in weekly time allocated for physical education in countries around the globe. For example, weekly time for physical education of secondary school students in Bangladesh (180 min) is reportedly higher than in Peru (90 min)^28^. Research is needed to understand whether physical education classes are designed to facilitate physical activity and/or how much time students actually spend in physical activity during physical education classes. It is also important to understand how physical education lessons can help the students to develop skills so that they can be more active both inside and outside of school. This information can help in designing a physical education curriculum with balanced components of physical activity and physical education lessons on other health and wellbeing so that the students can develop a healthy lifestyle. Opportunities for quality physical education should be equitable and inclusive, and available for all children regardless their gender, disability status, socio-economic position, and cultural or religious backgrounds, and the delivery of physical education should be ensured for marginalised and vulnerable groups^19^.

The strengths of our study are the inclusion of a large number of countries around the globe, representing different world regions and income groups. All countries included in our study provided nationally representative data. We used the GSHS sample weighting to account for distribution of the population by age and sex in countries for whose data were analysed. Any potential skewness, by sex or age, in the observed data is unlikely to impact the weighted analysis results. All countries where GSHS was implemented, used a standardised data collection procedure. In all countries, a standardised questionnaire with the same survey items to assess physical activity and physical education class attendance was used, which facilitated our regional comparisons. We adjusted our estimates for several potential covariates to avoid possible confounding effects of these factors.

The findings of our study should be interpreted in light of its limitations. Data for our study were collected using self-reported questionnaire; these data are vulnerable to social desirability and recall bias. Unavailability of GSHS data from European and North American countries, some of the Latin/Central American and Asia and Pacific countries, limits the generalisability of the findings only to the GSHS participating countries. Although a standardised questionnaire was used in all participating countries, there is a lack of information on the reliability and validity of GSHS measures across different countries or cultures. Physical education classes can have different meanings and can constitute different components, including a knowledge-based curriculum component (i.e., lessons and discussions) and/or skill-based physical activity session, in different settings. We did not have any information on components of physical education classes across the participating countries. The cross‐sectional design of the study limits our ability to make any causal inferences from the association estimates. Some adolescents in our study may have had difficulties with understanding the questionnaire because of poor reading skills. In this study, we used data collected between 2007 and 2016, which may have biased the results because of the period effect.

## Conclusions

Our study suggests a positive association between regular participation in physical education classes and meeting the physical activity guidelines among children and adolescents around the globe regardless of sex or age group. The odds were lower in high- than low-income countries. The benefits of regular participation in physical education classes to enhance physical activity are universal across all WHO regions, with the highest being observed among adolescents from South East Asian countries. Even less frequent participation in physical education classes (i.e., 1–2 days a week) was related to higher odds of being sufficiently active in all but low-income countries, especially in boys. Thus, the findings support the importance of physical education for ensuring sufficient physical activity among school-going children and adolescents around the globe. Countries must not miss the opportunity to ensure schools deliver a daily or at least 3 days per week of well-designed physical education classes, which can play a vital role in creating active nations around the world.

## Supplementary Information

Supplementary Table S1.
