# Supplementary material for: Physical education class participation is associated with physical activity among adolescents in 65 countries
Source: Sci Rep. 2020 Dec 17;10:22128. doi: 10.1038/s41598-020-79100-9 (PMC7746694; doi:10.1038/s41598-020-79100-9)
Supplement: Supplementary file 1 — Supplementary Table S1. [file 41598_2020_79100_MOESM1_ESM.docx]

**Supplementary Table S1**: Pooled estimates of association between frequency of physical education classes and sufficient physical activity among adolescents aged 11–17 years, by World Health Organization regions, Global School-based Student Health Surveys, 2007–2016.

|  |  | **Physical education classes ≥3 days/week**  **and physical activity** | **Physical education classes 1–2 days/week**  **and physical activity** |
| --- | --- | --- | --- |
|  | **Category** | **OR^1^ (95% CI)** | **OR^1^ (95% CI)** |
| Boys | Africa | 2.41, 1.74–3.33 | 1.25, 0.97–1.60 |
|  | The Americas | 1.62, 1.33–1.97 | 1.29, 1.06–1.58 |
|  | Eastern Mediterranean | 2.09, 1.77–2.47 | 1.23, 1.01–1.51 |
|  | South East Asia | 3.29, 1.97–5.47 | 1.86, 0.92–3.79 |
|  | Western Pacific | 2.18, 1.80–2.63 | 1.25, 1.00–1.57 |
| Girls | Africa | 2.63, 1.63–4.26 | 1.41, 1.15–1.73 |
|  | The Americas | 1.25, 1.02–1.55 | 0.97, 0.82–1.14 |
|  | Eastern Mediterranean | 1.90, 1.54–2.35 | 1.07, 0.84–1.36 |
|  | South East Asia | 2.35, 1.90–2.90 | 1.73, 0.91–3.28 |
|  | Western Pacific | 2.68, 1.89–3.77 | 1.14, 0.97–1.33 |
| 11-14 | Africa | 2.95, 2.07–4.20 | 1.38, 1.03–1.84 |
| years | The Americas | 1.56, 1.25–1.94 | 1.29, 1.07–1.56 |
|  | Eastern Mediterranean | 2.10, 1.75–2.53 | 1.24, 1.06–1.44 |
|  | South East Asia | 3.03, 2.42–3.79 | 1.60, 0.95–2.69 |
|  | Western Pacific | 2.62, 2.05–3.36 | 1.20, 0.95–1.52 |
| 15-17 | Africa | 2.29, 1.60–3.30 | 1.24, 1.03–1.48 |
| years | The Americas | 1.53, 1.26–1.86 | 1.05, 0.85–1.29 |
|  | Eastern Mediterranean | 2.14, 1.81–2.53 | 1.26, 1.07–1.49 |
|  | South East Asia | 3.24, 1.57–6.67 | 1.94, 0.99–3.77 |
|  | Western Pacific | 2.20, 1.74–2.80 | 1.19, 1.01–1.41 |

^1^Reference being attending no physical education class. OR=odds ratio; CI=confidence intervals
